# Assembly and Transfer of Iron–Sulfur Clusters in the Plastid

**DOI:** 10.3389/fpls.2018.00336

**Published:** 2018-03-14

**Authors:** Yan Lu

**Affiliations:** Department of Biological Sciences, Western Michigan University, Kalamazoo, MI, United States

**Keywords:** iron–sulfur cluster, cysteine desulfurase, sulfur transferase, iron–sulfur scaffold complex, iron–sulfur carrier protein

## Abstract

Iron-Sulfur (Fe-S) clusters and proteins are essential to many growth and developmental processes. In plants, they exist in the plastids, mitochondria, cytosol, and nucleus. Six types of Fe-S clusters are found in the plastid: classic 2Fe-2S, NEET-type 2Fe-2S, Rieske-type 2Fe-2S, 3Fe-4S, 4Fe-4S, and siroheme 4Fe-4S. Classic, NEET-type, and Rieske-type 2Fe-2S clusters have the same 2Fe-2S core; similarly, common and siroheme 4Fe-4S clusters have the same 4Fe-4S core. Plastidial Fe-S clusters are assembled by the sulfur mobilization (SUF) pathway, which contains cysteine desulfurase (EC 2.8.1.7), sulfur transferase (EC 2.8.1.3), Fe-S scaffold complex, and Fe-S carrier proteins. The plastidial cysteine desulfurase-sulfur transferase-Fe-S-scaffold complex system is responsible for *de novo* assembly of all plastidial Fe-S clusters. However, different types of Fe-S clusters are transferred to recipient proteins via respective Fe-S carrier proteins. This review focuses on recent discoveries on the molecular functions of different assembly and transfer factors involved in the plastidial SUF pathway. It also discusses potential points for regulation of the SUF pathway, relationships among the plastidial, mitochondrial, and cytosolic Fe-S assembly and transfer pathways, as well as several open questions about the carrier proteins for Rieske-type 2Fe-2S, NEET-type 2Fe-2S, and 3F-4S clusters.

## Introduction

Iron–Sulfur (Fe-S) clusters are sulfide (S^2−^)-linked di-iron, tri-iron, or tetra-iron clusters found in metalloproteins. Iron (Fe) is a transition metal, which can form cations with an incomplete *d* subshell. This property makes Fe show a variable valency (e.g., Fe^2+^ and Fe^3+^) and the ability to form coordination units, such as Fe-S clusters. Depending on the ligands, organic structures, and protein folds, the redox potential of Fe-containing cofactors may range between −650 and +450 mV (Beinert, 2000).

Due to the varying redox potential of Fe, Fe-S clusters have the ability to transfer electrons, especially when they are arranged sequentially with individual distances of <14 Å (Balk and Schaedler, 2014). Fe-S clusters are best known for participating in oxidation-reduction reactions in photosynthetic electron transport in thylakoid membranes and respiratory electron transport in the inner mitochondrial membrane (Johnson et al., 2005; Balk and Pilon, 2011; Couturier et al., 2013). Examples of Fe-S proteins involved in photosynthetic electron transport include the photosynthetic electron transfer C (PetC) protein in the cytochrome *b*_6_*f* complex, Photosystem I (PSI) core subunits PsaA, PsaB, and PsaC, and ferredoxins (Balk and Pilon, 2011). Examples of Fe-S complexes involved in respiratory electron transport include NADH dehydrogenase (Complex I, EC 1.6.99.3), succinate dehydrogenase (Complex II, EC 1.3.5.1), and cytochrome *bc*1 complex (Complex III, EC 1.10.2.2) (Couturier et al., 2013).

## Common types of Fe-S clusters and examples of Fe-S proteins in the plastid

**Figure 1:**
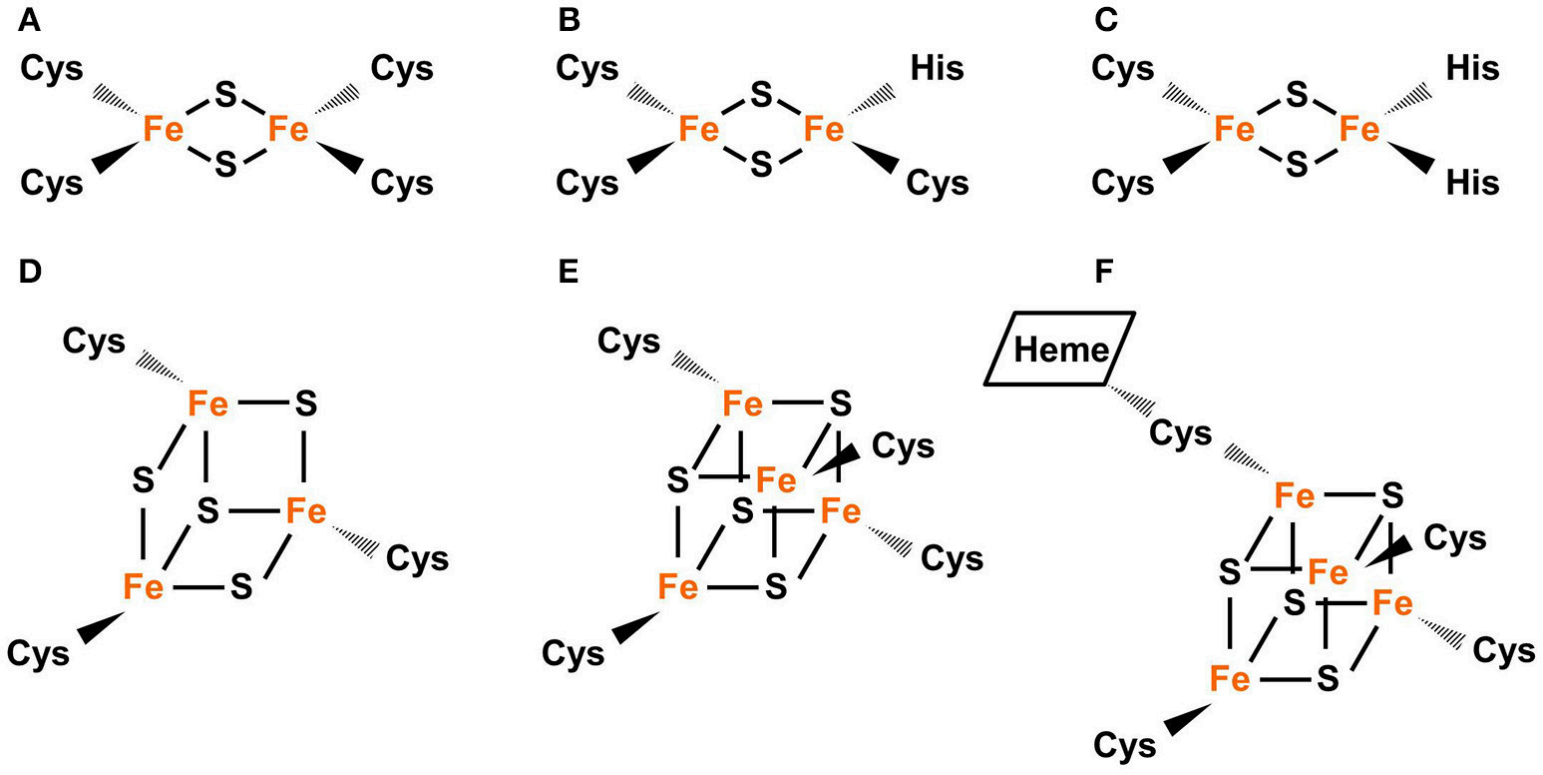
Common types of Fe-S clusters found in the plastid. **(A)** Classic 2Fe-2S coordinated by four Cys residues, as in the plant-type Fd (ferredoxin). **(B)** NEET-type 2Fe-2S coordinated by three Cys and one His residues, as in NEET. **(C)** Rieske-type 2Fe-2S coordinated by two Cys and two His residues, as in PetC (photosynthetic electron transfer **C)**. **(D)** 3Fe-4S coordinated by three Cys residues as in Fd-GOGATs (ferredoxin-dependent Gln oxoglutarate aminotransferases). **(E)** 4Fe-4S coordinated by four Cys residues, as in PsaA, PsaB, and PsaC (Photosystem I proteins **A–C)**. **(F)** 4Fe-4S coordinated by four Cys residues with a thiolate ligand serving also for siroheme, as in NiR (nitrite reductase) and SiR (sulfite reductase). In Fe-S clusters, the oxidation state of Fe could be +2 or +3 while the oxidation state of S is −2.

Common types of Fe-S clusters found in the plastid include (1) classic 2Fe-2S coordinated by four cysteine (Cys) residues; (2) NEET-type 2Fe-2S coordinated by three Cys and one His residues; (3) Rieske-type 2Fe-2S coordinated by two Cys and two His residues; (4) 3Fe-4S coordinate by three Cys residues; (5) 4Fe-4S coordinated by four Cys residues (or three Cys residues and the hydroxide group from water); and (6) 4Fe-4S coordinated by four Cys residues with one Cys cross-bridging a siroheme (Figure 1) (Johnson et al., 2005; Balk and Pilon, 2011; Couturier et al., 2013; Balk and Schaedler, 2014). 3Fe-4S and 4Fe-4S clusters are cubane-type clusters. The three 2Fe-2S clusters are rhombic-type clusters, containing the same 2Fe-2S core. Therefore, the relative abundances of the three rhombic-type clusters depend not on the rate of cluster assembly, but on the availability of appropriate carriers and recipient proteins.

The plant-type ferredoxin (Fd) is a classic 2Fe-2S protein. It is a small and soluble protein with four conserved Cys residues capable of ligating one 2Fe-2S cluster (Hanke and Mulo, 2013). In the chloroplast stroma, Fd acts as a mobile electron carrier during photosynthetic electron transport, carrying electrons from PSI to PSI-associated Fd-NADP^+^ reductase, for the reduction of NADP^+^ to NADPH (Hase et al., 2006). The plant-type Fd also serves as an electron donor to a number of chloroplast stromal proteins, such as nitrite reductase (NiR, EC 1.7.7.1), sulfite reductase (SiR, EC 1.8.7.1), and Fd-dependent glutamine:2-oxyoglutarate aminotransferases (Fd-GOGATs, EC 1.4.7.1) (Hase et al., 2006). Whether Fd serves as an electron carrier or electron donor, the electron is actually carried by the 2Fe-2S cluster within the protein (Hase et al., 2006).

One example of NEET-type 2Fe-2S proteins is At-NEET, a CDGSH motif-containing *Arabidopsis thaliana* protein dually targeted to the chloroplast stroma and mitochondria (Nechushtai et al., 2012; Su et al., 2013). The CDGSH motif contains a 16-amino acid consensus sequence (C-X-C-X_2_-[S/T]-X_3_-P-X-C-D-G-[S/A/T]-H, where the three Cys [C^74^, C^76^, and C^85^] residues and one His [H^89^] residue for NEET-type 2Fe-2S cluster coordination are underlined). The recombinant At-NEET homodimer coordinates two labile 2Fe-2S clusters, which are readily transferred to apo Fd in *in vivo* assays (Nechushtai et al., 2012). Therefore, Nechushtai et al. (2012) proposed that AT-NEET may serve as a NEET-type 2Fe-2S carrier for plastidial and mitochondrial Fe-S assembly and transfer pathways. Compared to classic 2Fe-2S, NEET-type 2Fe-2S is relatively unstable due to its atypical coordination with three Cys and one His residues (Wiley et al., 2007). Protonation of the ligating His residue could trigger cluster release, indicating that His ligation is also important for the pH lability of NEET-type 2Fe-2S (Wiley et al., 2007).

PetC is an example of Rieske-type 2Fe-2S proteins. The Rieske-type 2Fe-2S cluster in PetC is essential to photosynthetic electron transport. It accepts electrons from plastoquinol and transfers to the heme Fe of the cytochrome *f* protein (Madueño et al., 1992). Riesk-type 2Fe-2S proteins contain a Rieske-type 2Fe-2S-binding domain (CXHXGCX_12−44_CXCH, where the two Cys and two His residues for cluster coordination are underlined) (Link, 1999). The asymmetric coordination pattern (Figure 1C) of Rieske-type 2Fe-2S results in distinctive redox and spectroscopic properties (Kounosu et al., 2004). Compared to classic 2Fe-2S, Rieske-type 2Fe-2S has a relatively positive midpoint redox potential and its visible spectrum is red-shifted (Mason and Cammack, 1992).

Fd-GOGATs are examples of 3Fe-4S proteins. Plants have two Fd-GOGAT isoforms: Fd-GOGAT1 and Fd-GOGAT2 (Coschigano et al., 1998). Fd-GOGAT1 is expressed in leaf chloroplasts and its primary role is photorespiration and nitrogen assimilation in leaves; Fd-GOGAT2 is expressed in root plastids and its primary role is nitrogen assimilation in roots (Coschigano et al., 1998). Fd-GOGATs function via non-covalent binding of Fd and subsequent delivery of reducing equivalents from Fd to FMN (another cofactor) via the 3Fe-4S cluster (van den Heuvel et al., 2002). Both FMN and 3Fe-4S are located in the catalytic centers of Fd-GOGATs (van den Heuvel et al., 2002).

PSI core proteins PsaA, PsaB, and PsaC are examples of 4Fe-4S proteins. PSI has three 4Fe-4S clusters, each coordinated by four Cys residues. One is known as F_X_, which is bound to the PsaA/PsaB heterodimer. The other two are known as F_A_ and F_B_, both are bound to PsaC (Saenger et al., 2002). These 4Fe-4S clusters are essential to photosynthetic electron transport: they serve as sequential electron carriers (F_X_→ F_A_→ F_B_) within PSI.

NiR and SiR, two enzymes catalyzing the six electron reduction of nitrite and sulfite respectively (Raux-Deery et al., 2005), are siroheme 4Fe-4S proteins. The active site of these enzymes has a siroheme attached to the 4Fe-4S cluster via a Cys residue (Crane et al., 1995; Crane and Getzoff, 1996). Therefore, the siroheme 4Fe-4S cluster is central to the reductive activity of NiR and SiR. The insertion of Fe into siroheme is carried out by sirohydrochlorin ferrochelatase (EC 4.99.1.4), which contains a 2Fe-2S cluster (Saha et al., 2012).

In Fe-S clusters, Fe^2+^ or Fe^3+^ is combined with sulfide (S^2−^). The valency of the entire cluster could be indicated with square brackets (e.g., [2Fe-2S]^2+^, in which both Fe ions exist as Fe^3+^). After S^2−^ is incorporated into the cluster, it does not undergo redox transitions. However, Fe^3+^ in the cluster may be reduced to Fe^2+^ and *vice versa*. For example, [2Fe-2S]^2+^ may receive an electron and become reduced to [2Fe-2S]^1+^ and [2Fe-2S]^1+^ may lose an electron and become oxidized to [2Fe-2S]^2+^.

Different types of Fe-S clusters are inter-convertible. [4Fe-4S]^2+^ (a common oxidation state of 4Fe-4S) could be converted to [2Fe-2S]^2+^ (a common oxidation state of 2Fe-2S) via oxidative cleavage (Holm and Lo, 2016). For example, [4Fe-4S]^2+^-containing fumarate and nitrate reduction regulatory protein (FNR) is a DNA-binding homodimer under anaerobic conditions (Zhang et al., 2012). Under high oxygen, the [4Fe-4S]^2+^ cluster is quickly converted to a classic [2Fe-2S]^2+^ cluster, with the release of one Fe^3+^ ion, one Fe^2+^ ion, two S^2−^ ions, and a superoxide ion (${\text{O}}_{2}^{-}$), via a two-step process (Crack et al., 2008; Zhang et al., 2012). In the first step, [4Fe-4S]^2+^ is oxidized by one electron from molecular oxygen, producing an intermediate [3Fe-4S]^1+^, an Fe^2+^ ion, and a ${\text{O}}_{2}^{-}$ ion. In the second step, the intermediate [3Fe-4S]^1+^ converts spontaneously to [2Fe-2S]^2+^, releasing an Fe^3+^ ion and two S^2−^ ions. This two-step conversion causes the transition of FNR from dimer to monomer and the loss of DNA-binding ability. The [4Fe-4S]^2+^-to-[2Fe-2S]^2+^ conversion could be reversed via reductive coupling. For example, two [2Fe-2S]^2+^ clusters in FNR could receive two electrons and be reverted to [4Fe-4S]^2+^ after incubation with DTT under anaerobic conditions (Zhang et al., 2012).

Fe-S clusters are sensitive to oxygen and reactive oxygen species (ROS) (Couturier et al., 2013; Balk and Schaedler, 2014) and the assembly of Fe-S clusters is influenced by the availability of Fe and S (Vigani et al., 2009). The inter-convertibility among different types of Fe-S clusters may represent a route to repair damaged Fe-S clusters and/or a regulatory process in response to changes in cellular and external environments, especially redox status. As discussed below, loss-of-function mutations in a specific plastidial Fe-S carrier protein do not result in uniform reductions in the levels of different types of plastidial Fe-S clusters. These observations suggest that cluster inter-conversion may not be sufficient to compensate for the loss of a specific Fe-S carrier protein.

## Fe-S assembly and transfer pathways in plants

**Figure 2:**
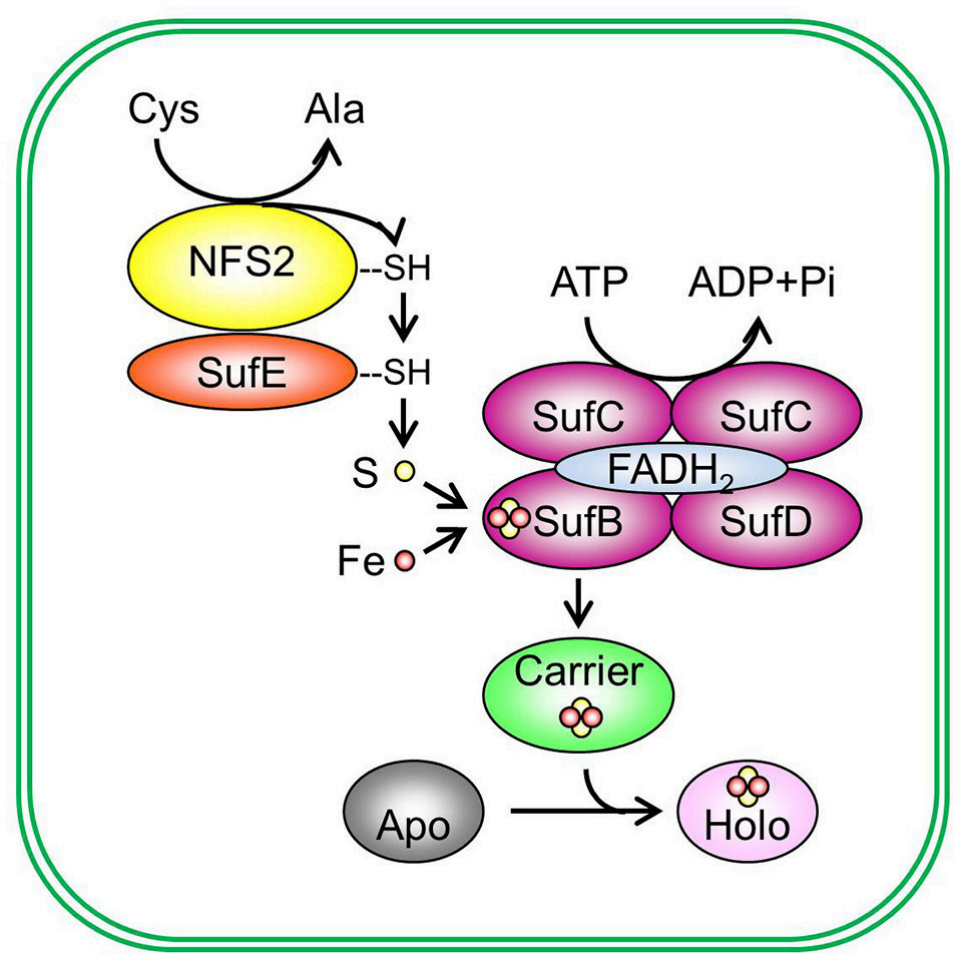
*De novo* assembly and transfer of Fe-S clusters in the plastid. In the plastid, Cys desulfurase NFS2 (nitrogen fixation S-like 2) removes sulphane (S^0^) from Cys and converts cysteine (Cys) to alanine (Ala). Sulphane (S^0^) is transferred from Cys desulfurase to sulfur transferase SufE (sulfur mobilization protein E) and then to SufB (sulfur mobilization protein B) of the SufBC_2_D scaffold complex. On the SufBC_2_D scaffold complex, whose function requires cofactor FADH_2_, sulphane (S^0^) is reduced to sulfide (S^2−^) and is incorporated in Fe-S clusters. The SufBC_2_D complex has ATPase activity, thus coupling ATP hydrolysis with the formation of Fe-S clusters. The source of Fe is not yet known. The newly assembled Fe-S cluster is then transferred to a carrier protein, which delivers the Fe-S cluster to recipient apoproteins and converts recipient apoproteins (Apo) into holoproteins (Holo). Plastidial sulfur transferases include SufE1, SufE2, and SufE3. The Suf scaffold complex is composed of three different proteins: SufB, sulfur mobilization protein C (SufC), and sulfur mobilization protein D (SufD), primarily in a 1:2:1 (BC_2_D) ratio. Potential plastidial Fe-S carriers include sulfur mobilization protein A1 (SufA1), nitrogen fixation subunit U 1, 2, and 3 (NFU1, NFU2, and NFU3), high chlorophyll fluorescence 101 (HCF101), plastidial Clusters of Orthologous Groups 0354 protein (COG0354p), and glutaredoxin S14 and S16 (GRXS14 and GRXS16). Pi, inorganic phosphate.

The Fe-S assembly and transfer process can be separated into two sequential steps (Figure 2). The first step is the assembly of Fe-S clusters on a scaffold complex by Cys desulfurase (EC 2.8.1.7), with the help from sulfur transferase (EC 2.8.1.3). The second step is the transfer of Fe-S clusters from the scaffold complex to recipient proteins via carrier proteins. The initial identification of proteins involved in Fe-S assembly was from the analysis of proteins required for nitrogen fixation in Gram-negative bacterium *Azotobacteria vinelandii* (Frazzon and Dean, 2003; Dos Santos et al., 2004). The work on the synthesis of nitrogenase (molybdenum [Mo])-Fe-S cofactors led to the discovery of the NIF (nitrogen fixation) system and established the requirement of Cys desulfurase, sulfur transferase, and Fe-S scaffold protein(s) during Fe-S assembly (Frazzon and Dean, 2003). While the NIF system specifically deals with nitrogenase cofactor synthesis in nitrogen-fixing bacteria (Jacobson et al., 1989; Frazzon and Dean, 2003), the assembly of Fe-S clusters for other Fe-S proteins in bacteria is carried out by the iron–sulfur cluster (ISC) system and the sulfur mobilization (SUF) system (Takahashi and Tokumoto, 2002; Outten et al., 2004). The bacterial ISC system contains the following proteins: Cys desulfurase IscS, scaffold protein IscU, DnaK-like chaperone HscA (heat shock cognate protein A), DnaJ-like co-chaperone HscB (heat shock cognate protein B), and possibly Fd (Ayala-Castro et al., 2008; Roche et al., 2013). The bacterial SUF system contains two complexes: SufSE (where SufS is Cys desulfurase and SufE is sulfur transferase), and SufBC_2_D (the scaffold complex). The bacterial ISC and SUF systems have different tolerance to oxidative stress (Dai and Outten, 2012). In SufS, the catalytic active Cys residue is deeply buried and thus not easily accessed by O_2_ or H_2_O_2_ (Lima, 2002). On the contrary, the catalytic active Cys residue in IscS is exposed to the surface of the protein (Cupp-Vickery et al., 2003). Therefore, it was proposed that the ISC system is the housekeeping system for Fe-S assembly and the SUF system is adapted to assemble Fe-S clusters under Fe or S starvation, or oxidative stress (Outten et al., 2004). Consistent with this hypothesis, SufS was found to express under Fe starvation, low S availability, and oxidative stress conditions (Outten et al., 2004). If readers are interested in detailed information on the regulation of bacterial ISC and SUF systems and the functions of individual Fe-S assembly proteins, they may refer to reviews on bacterial Fe-S assembly pathways, for example, Ayala-Castro et al. (2008) and Roche et al. (2013), and the references therein.

There are three Fe-S assembly and transfer pathways in the plant cell: the SUF pathway in the plastid, the ISC pathway in the mitochondrion, and the cytosolic iron–sulfur assembly (CIA) pathway (Balk and Pilon, 2011; Couturier et al., 2013; Balk and Schaedler, 2014). These pathways supply Fe-S clusters to the plastids, mitochondria, and cytosol plus nucleus, respectively. It is worth mentioning that the ISC pathway in plants evolved from the bacterial ISC system, which has poor tolerance to oxidative stress, and the SUF pathway in plants evolved from the bacterial SUF system, which has higher tolerance to oxidative stress (Takahashi and Tokumoto, 2002; Outten et al., 2004). Oxygenic phototrophs such as cyanobacteria and plants generate oxygen in photosynthetic cells or tissues. Therefore, it is likely that cyanobacteria and plants inherited the bacterial SUF system to tolerate the high oxidative environment at the site of photosynthesis. The plastidial SUF pathway appears to be independent from the mitochondrial ISC pathway and the CIA pathway (Van Hoewyk et al., 2007; Bernard et al., 2013). However, the CIA pathway requires the function of the mitochondrial ISC pathway: the sulfide compound from the ISC way is exported from the mitochondrion to the cytosol via ATM3 (ATP binding cassette transporter of the mitochondrion 3) and it serves as the S source for the CIA pathway (Kushnir et al., 2001; Kim et al., 2006; Bernard et al., 2009). This review focuses on recent discoveries on the molecular functions of different types of proteins involved in the plastidial SUF pathway. Detailed information about the ISC and ICA pathways in plants could be found in the following excellent reviews: Balk and Pilon (2011), Couturier et al. (2013), and Balk and Schaedler (2014).

### Cys desulfurase

Cys desulfurase removes S from Cys and converts Cys to alanine (Ala) (Figure 2) (Zheng et al., 1993, 1994; Zheng and Dean, 1994). The newly released sulphane S (S^0^) is bound to the active site of Cys desulfurase in the form of persulfide (R-S-S^0^H). The bound sulphane (S^0^) is then transferred in the same form to the Fe-S scaffold complex with the help from sulfur transferase.

Cys desulfurases can be classified into two groups according to their sequences and structures (Mihara et al., 1997; Mihara and Esaki, 2002; Roret et al., 2014). The two groups differ in the β-hairpin loop and the extended lobe containing the catalytic Cys residue. In group I, the extended loop lacks the β-hairpin structure and it is long and sufficiently flexible to transfer S^2−^ to sulfur transferase (Kaiser et al., 2000). Thus the sulfur transferase associated with a group I Cys desulfurase does not need to be flexible to acquire S^2−^. On the contrary, group II Cys desulfurases have a β-hairpin structure constraining the catalytic site and their extended loop is short and insufficiently flexible (Mihara et al., 2002; Outten et al., 2003; Singh et al., 2013). Therefore, the sulfur transferase associated with a group II Cys desulfurase needs additional flexibility to acquire S^2−^ (Kim and Park, 2013).

**Figure 3:**
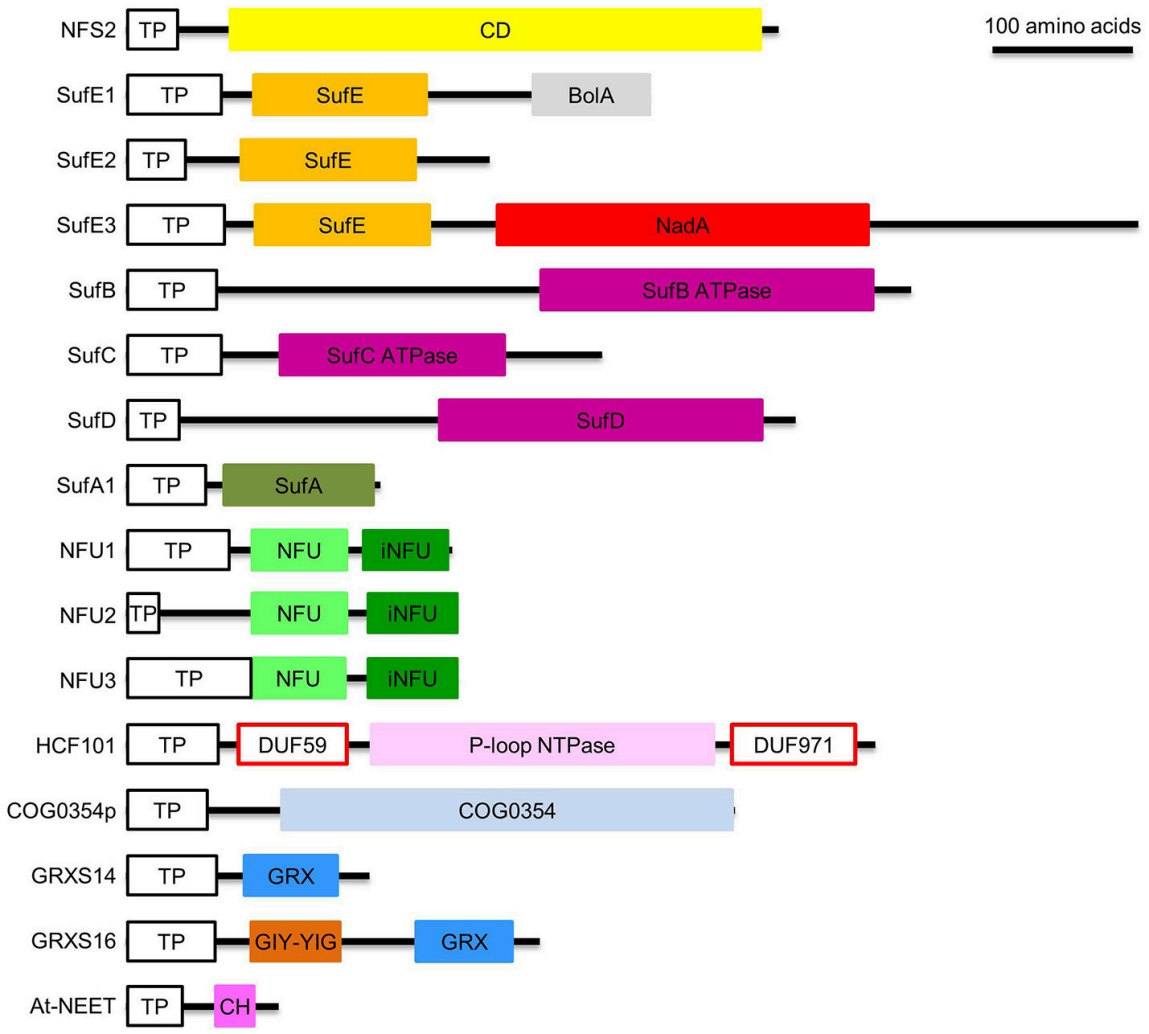
Domain composition of proteins involved in *de novo* assembly and transfer of Fe-S clusters in the plastid. ATPase, adenosine triphosphatase; BolA, DNA-binding transcriptional regulator BolA; CD, Cys desulfurase; CH, CDGSH motif; COG0354, Clusters of Orthologous Groups 0354 protein; COG0354p, plastidial Clusters of Orthologous Groups 0354 protein; DUF59 and DUF971; domain of unknown function 59 and 971; GIY-YIG, GlyIleTyr-TyrIleGly; GRX, glutaredoxin; GRXS14 and GRXS16, glutaredoxin S14 and S16; HCF101, high chlorophyll fluorescence 101; iNFU, redox-inactive nitrogen fixation subunit U; NadA, quinolinate synthase A; NFU, nitrogen fixation subunit U; NFU1, NFU2, and NFU3, nitrogen fixation subunit U 1, 2, and 3; NFS2, nitrogen fixation S-like 2; P-loop NTPase, P-loop nucleotide phosphatase; SufA1, SufB, SufC, SufD, SufE1, SufE2, and SufE3, sulfur mobilization protein A1, B, C, D, E1, E2, and E3; TP, transit peptide. Note that SufE1 and At-NEET are dually targeted to plastids and mitochondria. Bar = 100 amino acids.

Plants have three Cys desulfurases: nitrogen fixation S-like 1 (NFS1/NifS1, referred to as NFS1 hereafter) (Frazzon et al., 2007), nitrogen fixation S-like 2/chloroplastic nitrogen fixation S-like/chloroplastic sulfur mobilization protein S (NFS2/CpNifS/CpSufS, referred to as NFS2 hereafter) (Pilon-Smits et al., 2002; Ye et al., 2005; Van Hoewyk et al., 2007), and abscisic acid deficient 3 (ABA3) (Heidenreich et al., 2005). Among them, ABA3 is involved in Mo cofactor sulfuration but not in Fe-S assembly (Bernard et al., 2013). NFS1 is a group I Cys desulfurase (Mihara et al., 1997; Mihara and Esaki, 2002) and it is targeted to the mitochondrion (Kushnir et al., 2001). NFS2 is a group II Cys desulfurase (Mihara et al., 1997; Mihara and Esaki, 2002) and it is located to the chloroplast (Table 1) (Léon et al., 2002; Pilon-Smits et al., 2002). Full-length NFS2 has a 35-amino acid plastid transit peptide and a C-terminal Cys desulfurase domain (Figure 3) (Pilon-Smits et al., 2002). Gel filtration of the purified NFS2 protein showed this protein forms a dimer (Table 2) (Pilon-Smits et al., 2002; Ye et al., 2005). Recombinant NFS2 demonstrated Cys desulfurase activity toward Cys and seleno-Cys lyase activity toward seleno-Cys, with a much high activity on seleno-Cys (Pilon-Smits et al., 2002). Absorption spectrum indicated the presence of a pyridoxal 5′-phosphate cofactor in the purified protein (Pilon-Smits et al., 2002). NFS2 silencing via RNA interference (RNAi) is lethal (Van Hoewyk et al., 2007). Immunoblot analysis showed that the levels of Fe-S proteins representing different types of plastidial Fe-S clusters were reduced in the RNAi lines. However, mitochondrial Fe-S proteins and respiration were not affected in the RNAi lines. Taken together, these data indicate that NFS2 is required in *de novo* assembly of all plastidial Fe-S clusters and that the plastidial and mitochondrial Fe-S assembly pathways operate independently (Van Hoewyk et al., 2007).

**Table 1:** Proteins involved in *de novo* assembly and transfer of Fe-S clusters in the Arabidopsis plastid.

**Functional category**	**Protein name**	**Locus ID**	**Activity of purified recombinant protein**	**Phenotype of loss-of-function mutants**	**References**
Cys desulfurase	NFS2	At1g08490	Cys desulfurase activity	Seedling lethal	Pilon-Smits et al., 2002; Ye et al., 2005; Van Hoewyk et al., 2007
Activation of Cys desulfurase; sulfur transferase	SufE1	At4g26500	Activates Cys desulfurase; complements *E. coli* ΔSufE mutant	Embryo lethal	Xu and Møller, 2006; Ye et al., 2006
Activation of Cys desulfurase; sulfur transferase	SufE2	At1g67810	Activates Cys desulfurase	Not yet described	Murthy et al., 2007
Activation of Cys desulfurase; sulfur transferase	SufE3	At5g50210	Activates Cys desulfurase; quinolinate synthase activity; complements *E. coli* ΔNadA mutant	Embryo lethal	Katoh et al., 2006; Murthy et al., 2007
Scaffold complex	SufB	At4g04770	ATPase activity; complements *E. coli* ΔSufB mutant	Embryo lethal (strong alleles); pale green and growth retardation (weak alleles)	Møller et al., 2001; Ahn et al., 2005; Xu et al., 2005; Nagane et al., 2010; Saini et al., 2010; Wollers et al., 2010; Hu et al., 2017a,b
Scaffold complex	SufC	At3g10670	ATPase activity; complements *E. coli* ΔSufC mutant	Embryo lethal	Xu and Møller, 2004; Saini et al., 2010; Wollers et al., 2010; Hu et al., 2017a
Scaffold complex	SufD	At1g32500	Fe acquisition	Seed abortion; reduced chlorophyll content; defects in plastid morphology	Xu and Møller, 2004; Hjorth et al., 2005; Saini et al., 2010; Wollers et al., 2010; Hu et al., 2017a
Carrier protein	SufA1	At1g10500	Carrier of classic 2Fe-2S	No visible phenotype	Abdel-Ghany et al., 2005; Yabe and Nakai, 2006
Carrier protein	NFU1	At4g01940	Complements yeast *isu1 nfu1* double mutant	Not yet described	Léon et al., 2003
Carrier protein	NFU2	At5g49940	Carrier of classic 2Fe-2S and 4Fe-4S; complements yeast *isu1 nfu1* double mutant	Pale green; growth retardation; reduced levels of 2Fe-2S and 4Fe-4S proteins	Léon et al., 2003; Touraine et al., 2004; Yabe et al., 2004; Gao et al., 2013; Hu et al., 2017a
Carrier protein	NFU3	At4g25910	Carrier of 3Fe-4S and 4Fe-4S	Pale green; growth retardation; reduced levels of 3Fe-4S and 4Fe-4S proteins	Léon et al., 2003; Nath et al., 2016, 2017
Carrier protein	HCF101	At3g24430	Carrier of 4Fe-4S	Seedling lethal (strong alleles); reduced levels of 4Fe-4S proteins	Lezhneva et al., 2004; Stöckel and Oelmuller, 2004; Schwenkert et al., 2010; Hu et al., 2017a
Carrier protein	COG0354p	At1g60990	Complements *E. coli* ΔYgfZ and ΔMiaB mutants	Not yet described	Waller et al., 2010, 2012
Carrier protein; regulation of redox status	GRXS14	At3g54900	Carrier of 2Fe-2S; complements yeast *grx5* mutant; activates SufE1 by deglutathionating	Defects in early seedling growth under oxidative stresses; increased protein carbonylation in the chloroplast; no growth defects in adult plants under normal conditions	Cheng and Hirschi, 2003; Cheng et al., 2006; Bandyopadhyay et al., 2008; Yadav et al., 2012; Couturier et al., 2014; Rey et al., 2017
Carrier protein; regulation of redox status	GRXS16	At2g38270	Carrier of 2Fe-2S; GIY-YIG endonuclease activity; complements yeast *grx5* mutant; activates SufE1 by deglutathionating	No growth defects in adult plants under normal conditions	Cheng and Hirschi, 2003; Bandyopadhyay et al., 2008; Liu et al., 2013; Couturier et al., 2014; Rey et al., 2017
Carrier protein; regulation of redox status	At-NEET	At5g51720	Carrier of NEET-type 2Fe-2S	Delayed growth and development, accelerated senescence, and elevated ROS under normal conditions; increased sensitivity to low Fe; reduced sensitivity to high Fe	Nechushtai et al., 2012; Su et al., 2013

**Table 2:** Protein-protein interactions among different plastidial Fe-S assembly and transfer factors.

	**NFS2**	**SufE1**	**SufE2**	**SufE3**	**SufB**	**SufC**	**SufD**	**SufA1**	**NFU1**	**NFU2**	**NFU3**	**HCF101**	**COG0354p**	**GRXS14**	**GRXS16**	**At-NEET**
NFS2	+	+	+	+				+								
SufE1	+					–		–						+	+	
SufE2	+															
SufE3	+															
SufB					+	+	+									
SufC					+	+	+									
SufD					+	+										
SufA1	+	–						+								
NFU1																
NFU2										+						
NFU3																
HCF101																
COG0354p																
GRXS14		+												+		
GRXS16		+													+	
At-NEET																+

### Sulfur transferase

The Cys desulfurase activity of NFS2 is activated by sulfur transferases, which accept the persulfide (R-S-S^0^H) from NFS2 and transfer it to the Fe-S scaffold complex (Figure 2). Plants have three sulfur transferases: sulfur mobilization protein E1, E2, and E3, i.e., SufE1, SufE2, and SufE3 (Table 1) (Xu and Møller, 2006; Ye et al., 2006; Murthy et al., 2007). Full-length SufE1 contains a transit peptide, a SufE domain, and a BolA domain (Figure 3) (Xu and Møller, 2006; Ye et al., 2006). *In silico* analysis of the transit peptide suggests that SufE1 is dual-targeted to plastids and mitochondria. This prediction was confirmed with confocal microscopic analysis of tobacco and Arabidopsis leaves transiently expressing the AtSufE1-YFP (At stands for *A. thaliana*; YFP stands for yellow fluorescent protein) fusion protein (Xu and Møller, 2006). AtSufE1 was able to complement the growth defects of ΔSufE *Escherichia coli*. *In vitro* Cys desulfurization assays demonstrated that AtSufE1 interacts with and activates both plastid-targeted NFS2 and mitochondrion-targeted NFS1 (Table 2) (Xu and Møller, 2006; Ye et al., 2006). Loss-of-function AtSufE1 mutants are embryo lethal, indicating that SufE1 and Fe-S cluster assembly in plastids and mitochondria are essential during embryo development (Xu and Møller, 2006; Ye et al., 2006).

The function of the BolA domain in SufE1 was intriguing. BolA proteins are known as morphogens, whose overexpression results in spherical cell morphology in *E. coli* (Aldea et al., 1988). Surprisingly, overexpression of the BolA domain of AtSufE1 showed no effects on the morphology of *E. coli* or Arabidopsis (Xu and Møller, 2006). Therefore, Xu and Møller (2006) proposed that the BolA domain in SufE1 is not functional. However, recent yeast-two-hybrid and bimolecular fluorescence complementation assays demonstrated that the BolA domain allows SufE1 to interact with monothiol glutaredoxins (GRXs), including plastid-targeted GRXS14 and GRXS16 (Couturier et al., 2014). In addition, *in vitro* experiments showed that the binding of GRXs promotes the deglutathionation of SufE1, and thereby facilitates the activation of NFS2 by deglutathionated SufE1 (Couturier et al., 2014). Taken together, the interactions between the SufE1 BolA domain and GRXs suggest possible redox regulation of SufE1 activity and Fe-S cluster assembly by GRXs.

Full-length SufE2 and SufE3 have a plastid transit peptide and a SufE domain (Figure 3). Confocal microscopic analysis of Arabidopsis protoplasts expressing GFP (green fluorescent protein)-tagged AtSufE2 and AtSufE3 confirmed their plastidial localization (Murthy et al., 2007). The SufE domain of AtSufE2 and AtSufE3 was found to interact with and activate the Cys desulfurase activity of NFS2 (Table 2) (Murthy et al., 2007). SufE3 has a C-terminal NadA domain. In bacteria, the *NadA* gene encodes quinolinate synthase A (EC 2.5.1.72), an enzyme required for NAD biosynthesis (Foster and Moat, 1980). Expression of AtSufE3 complemented the *E. coli* ΔNadA mutant, suggesting that SufE3 contains a functional NadA domain (Murthy et al., 2007). Consistent with this hypothesis, recombinant AtSufE3 demonstrated quinolinate synthase (QS) activity. The expression of the NadA domain of AtSufE3 alone could not complement the *E. coli* ΔNadA mutant, indicating that the SufE domain of SufE3 is required for QS activity. Apparently, QS activity requires the 4Fe-4S cluster in the NadA domain; and the SufE domain is responsible for reconstituting the cluster by interacting with NFS2 (Murthy et al., 2007).

The phenotype of loss-of-function AtSufE2 mutants has not yet been reported. Loss-of-function AtSufE3 mutants are embryo lethal (Murthy et al., 2007), suggesting that SufE3 is an essential protein. This is somewhat surprising because there are three SufE proteins in plants. It is possible that the BolA domain in SufE1 and the NadA domain in SufE3 make the two proteins irreplaceable (Murthy et al., 2007). Differential expression could be another reason why the three SufE proteins cannot complement each other (Murthy et al., 2007). For example, SufE1 and SufE3 are expressed in vegetative tissues while SufE2 is only expressed in pollen. Furthermore, SufE1 is dual-targeted to both plastids and mitochondria while SufE2 and SufE3 are targeted to the plastid.

### The Fe-S scaffold complex

Persulfide (R-S-S^0^H) groups are transferred to the scaffold complex for Fe-S cluster assembly (Figure 2). During this step, sulphane (S^0^) accepts electrons from electron donors and is reduced to sulfide (S^2−^), the final form of S in Fe-S clusters. The scaffold complex of the SUF pathway is composed of three different proteins: sulfur mobilization protein B/non-intrinsic ABC protein 1 (SufB/NAP1; referred to as SufB hereafter; ABC stands for ATP binding cassette), sulfur mobilization protein C/non-intrinsic ABC protein 7 (SufC/NAP7; referred to as SufC hereafter), and sulfur mobilization protein D/non-intrinsic ABC protein 6 (SufD/NAP6; referred to as SufD hereafter), primarily in a 1:2:1 (BC_2_D) ratio (Xu et al., 2005; Hu et al., 2017a). However, other subcomplexes may form as well (Wollers et al., 2010; Roche et al., 2013; Hu et al., 2017a). Extensive protein-protein interactions have been observed among SufB, SufC, and SubD proteins (Table 2) (Xu and Møller, 2004; Xu et al., 2005; Hu et al., 2017a). Although all three proteins are required for *in vivo* assembly of Fe-S clusters (Table 1), formation of Fe-S clusters occurs directly on SufB (Saini et al., 2010). Each SufBC_2_D complex binds to one molecule of FADH_2_ via SufB (Wollers et al., 2010). FADH_2_ was thought to provide electrons to reduce sulphane (S^0^) to sulfide (S^2−^) (Wollers et al., 2010). However, Wollers et al. (2010) reported that FADH_2_ actually plays a role in reductive mobilization of Fe, which is equally important for the assembly of Fe-S clusters. Using lysed and intact chloroplasts, Takahashi et al. showed that plastidial formation of Fe-S clusters on the scaffold complex requires ATP and NADPH (Takahashi et al., 1986, 1991a,b). These observations were re-confirmed by the activation of the SUF pathway in cell lysates by the addition of ATP and NADPH (Saini et al., 2010). The hydrolysis of ATP is likely carried out by the SufBC_2_D complex, because the SufB and SufC subunits of this complex have ATPase activity (Xu and Møller, 2004; Xu et al., 2005). While ATP hydrolysis by the SufBC_2_D complex provides energy for the formation of Fe-S clusters (Xu and Møller, 2004; Xu et al., 2005), NADPH may serve as the reducing agent for redox cycling of FADH_2_/FAD (Saini et al., 2010). In addition to NADPH, another possible source of reducing agents is plant-type Fds. Because plant-type Fds participate in many plastidial reactions, direct evidence for their role in the SUF pathway is still lacking. However, mitochondrial Fd has been shown to be essential for *de novo* assembly of Fe-S clusters in yeast mitochondria (Takahashi and Nakamura, 1999).

The source of Fe for plastidial Fe-S assembly is not yet known. Universal Fe-storage protein ferritin was previously considered as a candidate to supply Fe to the Fe-S scaffold complex (Lobreaux and Briat, 1991; Briat and Lobréaux, 1997). However, the triple loss-of-function Arabidopsis mutant of three major chloroplastic ferritins FER1, FER3, and FER4 had normal photosynthetic apparatus, which is extremely dependent on proper assembly of Fe-S clusters (Ravet et al., 2009). These results suggest that chloroplastic ferritins do not constitute a major Fe pool for the plastidial SUF pathway. Interestingly, the triple mutant exhibited increased sensitivity to excess Fe, suggesting that chloroplastic ferritins may actually act as Fe scavengers.

Full-length SufB contains a plastid transit peptide and an adenosine triphosphatase (ATPase, EC 3.6.1.3) domain with degenerative Walker A (also known as P-loop) and Walker B motifs (Figure 3) (Xu et al., 2005). The plastidial localization of AtSufB was confirmed with confocal microscopic analysis of onion epidermal cells expressing the AtSufB-GFP fusion protein (Møller et al., 2001). AtSufB expression complemented the growth defects of *E. coli* ΔSufB mutant, suggesting that AtSufB is a functional SufB protein (Xu et al., 2005). However, the absence of either SufD or both SufC and SufD caused substantial reduction in the *in vivo* cluster-assembly ability of SufB (Hu et al., 2017a). These results indicate that SufC and SufD are required for *in vivo* Fe-S cluster formation on SufB. Recombinant AtSufB demonstrated Fe^2+^-stimulated ATPase activity (Xu et al., 2005). This is somewhat surprising because ATPase activity was not reported for prokaryotic SufB (Roche et al., 2013). SufB was previously classified as a non-intrinsic ABC protein; however its ATPase domain does not contain the conserved ABC signature motif. In addition, SufB is capable of forming homodimers (Table 2), which also is different from its prokaryotic counterparts. The strong loss-of-function AtSufB mutant is embryo lethal, suggesting that SufB and the SUF pathway are essential to embryogenesis (Nagane et al., 2010). Weak SufB alleles in Arabidopsis and *Nicotiana benthamiana* had pale green leaves and retarded growth, due to defects in chlorophyll biosynthesis and chloroplast development (Møller et al., 2001; Ahn et al., 2005; Nagane et al., 2010; Hu et al., 2017a,b). These data demonstrate that insufficient supply of Fe-S clusters in the plastid will lead to defects in chlorophyll biosynthesis, chloroplast development, growth retardation, and embryo development.

Full-length SufC contains a plastid transit peptide and an ATPase domain with degenerative Walker A and Walker B motifs and an ABC signature motif (Figure 3) (Xu and Møller, 2004). The plastidial localization of SufC was confirmed by microscopic analysis of tobacco leaves expressing the AtSufC-YFP fusion protein (Xu and Møller, 2004). AtSufC expression complemented the growth defects of *E. coli* ΔSufC mutant, suggesting that AtSufC is a functional SufC protein (Xu and Møller, 2004). Consistent with this observation, recombinant AtSufC demonstrated Mg^2+^-stimulated ATPase activity (Xu and Møller, 2004). Loss of AtSufC resulted in an embryo-lethal phenotype, suggesting that SufC and the SUF pathway are essential to embryogenesis (Xu and Møller, 2004). The RNAi lines of SufB demonstrated pale green leaves, because of their defects in chlorophyll biosynthesis (Hu et al., 2017a). Due to similarity to the ISC pathway, it was proposed that ATP hydrolysis by SufB and SufC induces conformational changes of the SufBC_2_D scaffold complex to release newly assembled Fe-S clusters (Markley et al., 2013). However, Saini et al. (2010) reported that the ATPase activity is instead necessary for Fe acquisition during *in vivo* assembly of Fe-S clusters.

Full-length SufD contains a plastid transit peptide and a SufD domain (Figure 3). The plastidial localization of SufD was confirmed by microscopic analysis of tobacco leaves expressing the AtSufD-YFP fusion protein (Xu and Møller, 2004). Unlike SufB or SufC, the SufD domain does not contain Walker A, Walker B, or the ABC signature motif. Saini et al. (2010) reported that SufD is not required for *in vivo* sulfur transfer from the NFS2-SufE system, but it is required during *in vivo* Fe acquisition and *in vivo* formation of Fe-S clusters on SufB (Saini et al., 2010). The loss-of-function mutation in the *SufD* gene resulted in seed abortion, reduced chlorophyll content, and defects in plastid morphology (Hjorth et al., 2005). Consistent with these observations, RNAi lines of this protein showed defective chlorophyll biosynthesis (Hu et al., 2017a). Taken together, these data indicate that SufD may have a general house-keeping role (e.g., chlorophyll biosynthesis and chloroplast development), instead of functioning only in embryogenesis (Hjorth et al., 2005).

### Fe-S carrier proteins

Fe-S clusters assembled on the SufBC_2_D scaffold complex are transferred to recipient proteins via carrier proteins (Figure 2). When exogenous Fe^2+^ and S^2−^ are supplied, Fe-S carrier proteins may serve as scaffold proteins for *in vitro* Fe-S cluster assembly. Examples of plastidial Fe-S carrier proteins include: sulfur mobilization protein A1/chloroplast ISC protein A1 (SufA1/CpIscA1, referred to as SufA1 hereafter) (Abdel-Ghany et al., 2005; Yabe and Nakai, 2006); nitrogen-fixation-subunit-U-type proteins NFU1, NFU2, and NFU3 (Léon et al., 2003; Touraine et al., 2004; Yabe et al., 2004; Gao et al., 2013; Nath et al., 2016, 2017); P-loop nucleotide phosphatase (NTPase) HCF101 (high chlorophyll fluorescence 101) (Lezhneva et al., 2004; Stöckel and Oelmuller, 2004; Schwenkert et al., 2010); plastidial-type COG0354 (COG0354p, COG stands for Clusters of Orthologous Groups; p stands for plastidial) (Waller et al., 2010, 2012); and monothiol GRXS14 and GRXS16 (S refers to the Ser residue in the CGFS active site motif of GRXs) (Table 1) (Cheng and Hirschi, 2003; Cheng et al., 2006; Bandyopadhyay et al., 2008; Yadav et al., 2012; Couturier et al., 2014).

#### SufA1

**Figure 4:**
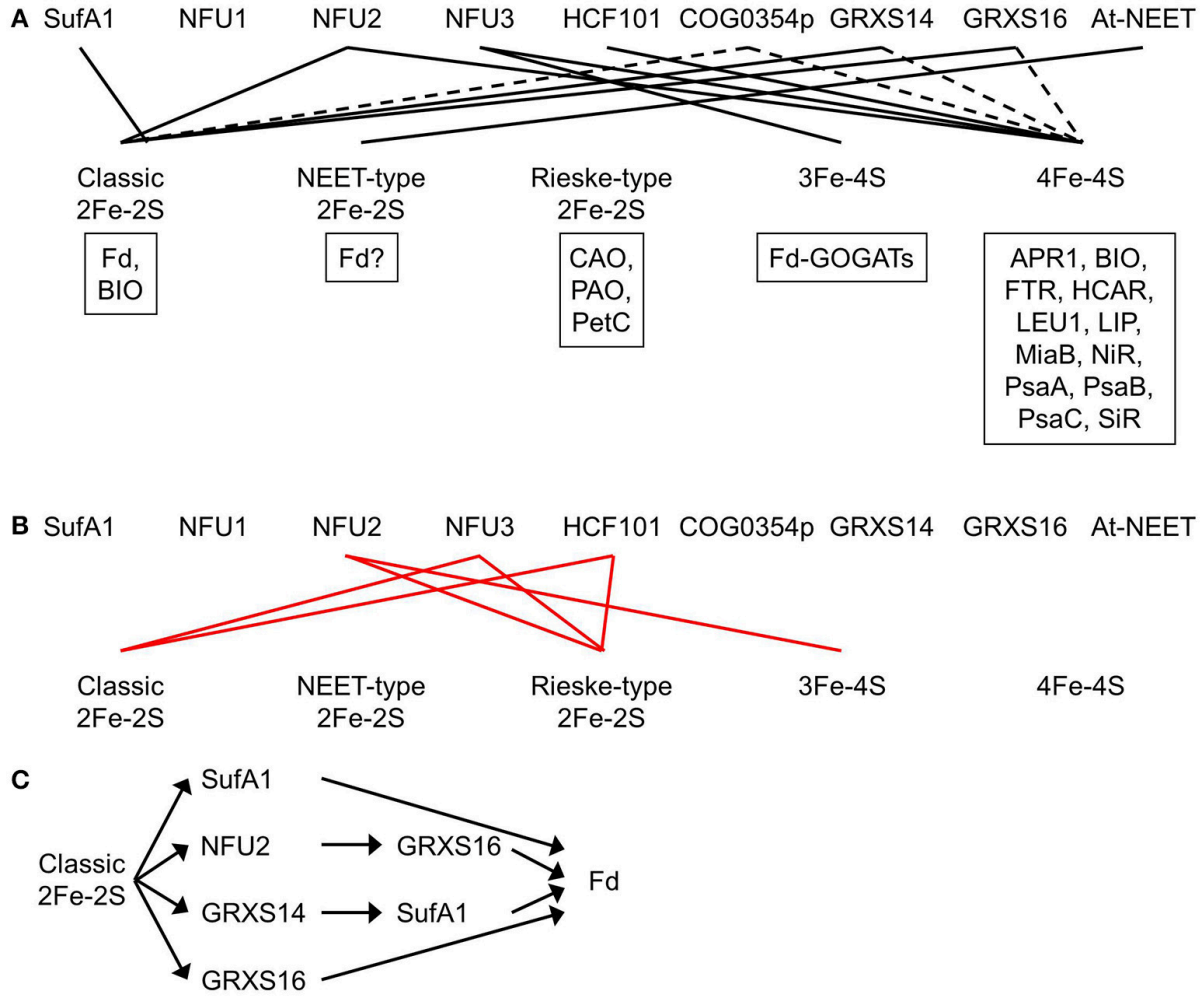
Plastidial Fe-S clusters and the corresponding Fe-S carrier proteins**. (A)** Plastidial Fe-S clusters, the corresponding Fe-S carrier proteins, and exemplar Fe-S proteins. Solid black lines indicate that the Fe-S carrier protein has been shown to act as a carrier for that type of Fe-S clusters. Dashed lines indicate that the Fe-S carrier protein could be a possible carrier for that type of Fe-S clusters. Exemplar Fe-S proteins containing that type of Fe-S clusters are shown in boxes. APR1, adenosine 5′-phosphosulfate reductase 1; At-NEET, *Arabidopsis thaliana* NEET; BIO, biotin synthase; CAO, chlorophyllide *a* oxygenase; COG0354p, plastidial Clusters of Orthologous Groups 0354 protein; Fd, ferredoxin; Fd-GOGATs, ferredoxin-dependent Gln oxoglutarate aminotransferases; FTR, ferredoxin-thioredoxin reductase; GRXS14 and GRXS16, glutaredoxin S14 and S16; HCAR, 7-hydroxymethyl chlorophyll *a* reductase; HCF101, high chlorophyll fluorescence 101; LEU1, 3-isopropylmalate isomerase 1; LIP, lipoic acid synthase; MiaB, isopentenyl-adenosine A37 tRNA methylthiolase; NFU1, NFU2, and NFU3, nitrogen fixation subunit U 1, 2, and 3; NiR, nitrite reductase; PetC, photosynthetic electron transfer C; PAO, pheophorbide *a* oxygenase; PsaA, PsaB, and PsaC, Photosystem I proteins A, B, and C; SiR, sulfite reductase. Note that BIO contains one classic 2Fe-2S and one 4Fe-4S. Recombinant At-NEET was able to transfer the NEET-type 2Fe-2S cluster to the plant-type Fd under *in vitro* conditions. However, it is not clear whether the plant-type Fd is able to serve as a recipient protein for the NEET-type 2Fe-2S cluster under *in vivo* conditions, because plant-type Fds are known as classic 2Fe-2S proteins. Therefore, a question mark is placed next to Fd in the box for exemplar NEET-type 2Fe-2S proteins. **(B)** Experimental evidence showed that NFU2 does not act as a carrier for Rieske-type 2Fe-2S and 3Fe-4S cluster and that NFU3 and HCF101 do not act as carriers for classic and Rieske-type 2Fe-2S clusters. Solid red lines indicate that the Fe-S carrier protein does not transfer that type of Fe-S clusters. **(C)** The transfer network of carrier proteins for classic 2Fe-2S clusters in the plastid. Fd is used as an example of recipient proteins.

The Arabidopsis genome encodes four SufA proteins: SufA1 is plastid-targeted (Figure 3; Table 1) while SufA2, SufA3, and SufA4 are mitochondrion-targeted (Yabe and Nakai, 2006). All four SufA proteins contain three conserved Cys residues: one in the GCXGXXY motif and the other two in the C-terminal C(G/S)CXSF motif (Yabe and Nakai, 2006). These conserved Cys residues may serve as ligands for Fe-S clusters. The plastidial localization of SufA1 was confirmed with a number of independent techniques, including confocal microscopic analysis of GFP-fusion protein, import assays, as well as chloroplast sub-fractionation and subsequent immunodetection (Abdel-Ghany et al., 2005). SufA1 is ubiquitously expressed in all the Arabidopsis tissues tested, with higher expression in green tissues such as leaves and flower stalks (Abdel-Ghany et al., 2005; Yabe and Nakai, 2006). Gel filtration analysis showed that SufA1 tends to form homodimers (Table 2). Recombinant SufA1 was capable of enhancing NFS2-mediated *in vitro* assembly of 2Fe-2S clusters on apo Fd (Abdel-Ghany et al., 2005). This observation suggests a possible interaction between SufA1 and NFS2 (Table 2), during which SufA1 may act as a scaffold protein for *in vitro* assembly of 2Fe-2S clusters. However, loss-of-function mutation in the *SufA1* gene does not cause any defect in plant growth and development or in the abundance of classic 2Fe-2S protein Fd (Yabe and Nakai, 2006). It is likely that SufA1 serves as a back-up carrier for classic 2Fe-2S (Figure 4A), when other classic 2Fe-2S carriers are insufficient, e.g., under stress conditions.

#### NFU1, NFU2, and NFU3

The Arabidopsis genome encodes five NFU proteins: NFU1, NFU2, and NFU3 are plastid-localized (Figure 3; Table 1) and NFU4 and NFU5 are mitochondrion-localized (Léon et al., 2003; Yabe et al., 2004; Gao et al., 2013; Nath et al., 2016). Their subcellular localization was confirmed with confocal microscopic analysis of Arabidopsis protoplasts expressing GFP-tagged NFU proteins (Léon et al., 2003). Reverse transcription-PCR analysis showed that NFU1 is expressed in flower stalks and siliques, NFU2 is expressed in flower stalks and leaves, and NFU3 is expressed in flower stalks, leaves, flowers, and roots (Léon et al., 2003).

Mature NFU1, NFU2, and NFU3 contain an N-terminal redox-active NFU domain with a conserved CXXC motif and a C-terminal redox-inactive NFU domain (Léon et al., 2003; Gao et al., 2013; Nath et al., 2016). The two Cys residues in the conserved CXXC motif of the redox-active NFU domain are used to coordinate Fe-S clusters. Both NFU1 and NFU2 were able to complement the growth defects of the yeast *isu1 nfu1* double mutant, suggesting that NFU1 and NFU2 are functional NFU proteins (Léon et al., 2003). The oligomerization and activity of recombinant NFU1 has not yet been tested *in vitro*. Recombinant NFU2 may form homodimers and homotetramers (Table 2) (Yabe et al., 2004; Gao et al., 2013). It served as a scaffold protein during *in vitro* reconstitution of classic 2Fe-2S and 4Fe-4S clusters (Figure 4A) and a carrier protein during subsequent *in vitro* transfer to apo GRXS16 and APR1 (adenosine 5′-phosphosulfate reductase 1, EC 1.8.99.2) proteins (Léon et al., 2003; Yabe et al., 2004; Gao et al., 2013). The unidirectional and intact transfer of classic 2Fe-2S from NFU2 to apo GRXS16 *in vitro* suggests that NFU2 may work upstream of GRXS16 during *in vivo* delivery of classic 2Fe-2S clusters to recipient proteins (Figure 4C) (Gao et al., 2013). NFU3 also is a functional NFU protein. Recombinant NFU3 served as a scaffold protein during *in vitro* reconstitution of 3Fe-4S and 4Fe-4S clusters (Figure 4A) (Nath et al., 2016).

The phenotype of loss-of-function mutants of NFU1 has not been described. Therefore, it is not clear which Fe-S cluster(s) NFU1 is able to carry. Loss-of-function mutations of the *NFU2* gene resulted in smaller and pale green leaves, a smaller plant size, as well as stunted growth and development (Touraine et al., 2004; Yabe et al., 2004). SDS-PAGE and immunoblot analysis showed that the levels of classic 2Fe-2S protein Fd, 4Fe-4S proteins PsaA, PsaB, and PsaC, and siroheme 4Fe-4S proteins NiR and SiR were largely reduced in the *nfu2* mutants while the level of Rieske-type 2Fe-2S protein PetC was substantially increased. Consistent with the immunoblot data, the activities of PSI and SiR were substantially reduced in the *nfu2* mutants while the activity of 3Fe-4S enzyme Fd-GOGAT was significantly increased. Taken together, these data demonstrate that NFU2 is required in the assembly and transfer of classic 2Fe-2S and 4Fe-4S clusters (Figure 4A). Excess Fe and S in the *nfu2* mutants could be used to make other Fe-S clusters such as Rieske-type 2Fe-2S and 3Fe-4S, hence, increased levels of Rieske-type 2Fe-2S protein PetC and 3Fe-4S proteins Fd-GOGATs. The elevated contents of PetC and Fd-GOGATs suggest that NFU2 does not serve as a carrier protein for Rieske-type 2Fe-2S or 3Fe-4S (Figure 4B).

Loss-of-function mutants of NFU3 exhibited retarded growth and development, along with smaller and pale green leaves and a smaller plant size (Nath et al., 2016, 2017). SDS-PAGE and immunoblot analysis showed that the levels of 4Fe-4S proteins PsaA, PsaB, and PsaC and 3Fe-4S proteins Fd-GOGATs were substantially reduced in the *nfu3* mutants while the levels of classic 2Fe-2S protein Fd and Rieske-type 2Fe-2S protein PetC were significantly increased. Consistent with the immunoblot data, PSI activity was nearly abolished in the *nfu3* mutants. Taken together, these results demonstrate that NFU3 is required in the assembly and transfer of 4Fe-4S and 3Fe-4S clusters (Figure 4A). Excess Fe and S in the *nfu3* mutants could be used to make other Fe-S clusters such as classic and Rieske-type 2Fe-2S, hence, increased levels of classic 2Fe-2S protein Fd and Rieske-type 2Fe-2S protein PetC. The elevated levels of Fd and PetC suggest that NFU3 does not serve as a carrier protein for classic or Rieske-type 2Fe-2S (Figure 4B).

#### HCF101

HCF101 is a plastid-localized P-loop NTPase with an N-terminal domain of unknown function 59 (DUF59) and a C-terminal domain of unknown function 971 (DUF971) (Figure 3; Table 1) (Lezhneva et al., 2004; Stöckel and Oelmuller, 2004; Schwenkert et al., 2010). The P-loop NTPase domain contains a Walker A motif (i.e., P-loop, CKGGVGKS), an A' motif (GARVGIFDADV), and a B motif (DYLVID) (Stöckel and Oelmuller, 2004; Schwenkert et al., 2010). Via site-directed mutagenesis, three Cys residues were found to be required for the assembly and/or stability of Fe-S clusters: C^128^, C^347^, and C^419^ (Schwenkert et al., 2010). C^347^ and C^419^ exist in the presence of a second Cys residue: C^339^X_7_C^347^ and C^414^X_4_C^419^. These Cys residues and motifs might be binding sites for Fe-S clusters. Analysis of Fe and S contents in reconstituted HCF101 showed that this protein may bind to one 4Fe-4S cluster per monomer (Schwenkert et al., 2010).

The plastidial localization of HCF101 was confirmed with import assays (Lezhneva et al., 2004). Recombinant HCF101 served as a scaffold protein during *in vitro* reconstitution of 4Fe-4S clusters and a carrier protein during subsequent *in vitro* transfer to apo LEU1 (3-isopropylmalate isomerase 1, EC 4.2.1.33) (Schwenkert et al., 2010). Complete loss-of-function mutations in the *HCF101* gene resulted in a seedling-lethal phenotype (Lezhneva et al., 2004; Stöckel and Oelmuller, 2004). In the *hcf101* mutants, the levels of 4Fe-4S proteins PsaA, PsaB, and PsaC were < 4% of those in the wild type (Lezhneva et al., 2004). In addition, the content of Fd-thioredoxin reductase (FTR, EC 1.8.7.2), another plastidial 4Fe-4S protein, was significantly reduced in the *hcf101* mutants. On the contrary, the amount of Rieske-type 2Fe-2S protein PetC was not reduced in the *hcf101* mutants and the amount of classic 2Fe-2S protein Fd was actually increased. Taken together, these data show that HCF101 is required in the assembly and transfer of 4Fe-4S (Figure 4A), but not classic or Rieske-type 2Fe-2S (Lezhneva et al., 2004) (Figure 4B). Because the amounts of representative 3Fe-4S proteins were not determined in the *hcf101* mutants, it is not clear whether HCF101 can serve as a carrier protein for 3Fe-4S.

#### COG0354p

COG0354 is a family of proteins classified in the Clusters of Orthologous Groups database (Waller et al., 2010). COG0354 proteins are found in all domains of life. IBA57 in yeast and YgfZ in *E. coli* are two well-studied COG0354 proteins. Mitochondrion-targeted IBA57 is required for the assembly of Fe-S clusters on mitochondrial aconitase (ACO, EC 4.2.1.3), as well as the activation of radical S-adenosylmethionine enzymes, such as isopentenyl-adenosine A37 tRNA methylthiolase (MiaB, EC 2.8.4.3), biotin synthase (BIO, EC 2.8.1.6), and lipoic acid synthase (LIP, EC 2.8.1.8) (Gelling et al., 2008). These four enzymes contain one 4Fe-4S, two 4Fe-4S, one classic 2Fe-2S plus one 4Fe-4S, and two 4Fe-4S clusters, respectively (Balk and Pilon, 2011). IBA57 physically interacted with mitochondrial Fe-S assembly protein 1 and 2 (ISA1 and ISA2) (Gelling et al., 2008), further demonstrating that COG0354 family proteins are part of the Fe-S assembly machinery. YgfZ, which is targeted to the mitochondria as well, is required for the activities of MiaB and some other Fe-S enzymes (Waller et al., 2010).

Plants have two COG0354 proteins: one is plastid-targeted (COG0354p; Figure 3; Table 1) and the other is mitochondrion-targeted (COG0354m) (Waller et al., 2010, 2012). Their subcellular localization was confirmed with import assays and confocal microscopic analysis of GFP fusion proteins (Waller et al., 2012). Both COG0354p and COG0354m were capable of complementing the growth defects of *E. coli* ΔYgfZ and ΔMiaB mutants (Waller et al., 2010, 2012). Similar to IBA57 and YgfZ, the activity of COG0354p and COG0354m is folate-dependent (Waller et al., 2012). Inactivation of COG0354m resulted in embryo lethality, indicating that COG0354m is an essential protein (Waller et al., 2012). The phenotype of loss-of-function mutants of COG0354p has not yet been described (Table 1). Therefore, it is not clear which Fe-S clusters are carried by COG0354p and COG0354m. However, because inactivation of IBA57 affects 4Fe-4S and classic 2Fe-2S enzymes (Gelling et al., 2008), it is possible that COG0354p and COG0354m are involved in the assembly and transfer of 4Fe-4S and/or classic 2Fe-2S clusters (Figure 4A).

#### GRXS14 and GRXS16

Higher plants have two plastidial GRXs: GRXS14 and GRXS16 (Figure 3; Table 1) (Bandyopadhyay et al., 2008). They were previously thought as activators of vacuolar H^+^/Ca^2+^ antiporters (Cheng and Hirschi, 2003). However, *in silico* analysis showed that full-length GRXS14 and GRXS16 contain a plastid transit peptide and a GRX domain with a CGFS active site motif (Cheng et al., 2006; Bandyopadhyay et al., 2008). The plastidial localization of GRXS14 and GRXS16 was confirmed by confocal microscopic analysis of GFP fusion proteins. After careful examination of the subcellular localization and physiological function, Cheng et al. (2006) reported that GRXS14 and GRXS16 do not act as activators of vacuolar H^+^/Ca^2+^ antiporters.

Both GRXS14 and GRXS16 were able to complement the growth defects of yeast *grx5* mutant, suggesting that these two proteins are functional GRXs (Bandyopadhyay et al., 2008). Analytical, absorption, and circular dichroism spectral data showed that GRXS14 and GRXS16 bind to one 2Fe-2S cluster per homodimer via the CGFS active site (Table 2) and the binding is assisted by the Cys residues of two glutathione molecules (Bandyopadhyay et al., 2008). In addition, apo GRXS14 served as the scaffold protein during Cys desulfurase-mediated *in vitro* 2Fe-2S cluster assembly and the 2Fe-2S cluster in GRXS14 could be readily transferred to apo Fd (Figure 4A) (Bandyopadhyay et al., 2008). Furthermore, a unidirectional and intact transfer of 2Fe-2S clusters from GRXS14 to SufA1 was observed, suggesting that GRXS14 may work upstream of SufA1 during *in vivo* delivery of 2Fe-2S to recipient proteins (Figure 4C) (Mapolelo et al., 2013). It should be noticed that introducing GRXS14 and GRXS16 into yeast *grx5* mutant also restores the activity of mitochondrial ACO (Bandyopadhyay et al., 2008). Because mitochondrial ACO requires a 4Fe-4S cluster to function, it is possible that GRXS14 and GRXS16 act as 4Fe-4S carriers as well (Figure 4A) (Rouhier et al., 2010).

In Arabidopsis, GRXS14 and GRXS16 have a similar expression profile: both are expressed in green photosynthetic tissues and flowers (Rey et al., 2017). Consistent with the expression profile, GRXS14 knockout lines and GRXS16 knockdown lines did not exhibit any phenotypic defects under standard growth conditions (Rey et al., 2017). However, the combination of GRXS14 knockout and GRXS16 knockdown resulted in retarded growth, suggesting that GRXS14 and GRXS16 have an overlapping role in the plastid.

Environmental constraints, such as oxidative stress, prolonged darkness, high irradiance, and high salt, change cellular redox status (Rey et al., 2017). Under oxidative stress, GRXS14 knockout lines displayed growth defects in early seedlings (Cheng et al., 2006). Prolonged darkness causes GRXS14 oxidation and reduced contents of proteins involved in maturation of Fe-S proteins (Rey et al., 2017). Under prolonged darkness, GRXS14 knockout lines exhibited accelerated chlorophyll loss. These data suggest that GRXS14 is important in protecting proteins again oxidative stress during early seedling growth and maintaining the chlorophyll level under prolonged darkness. Interestingly, GRXS14 overexpression lines demonstrated a reduce chlorophyll content under standard, high irradiance, and high salt conditions (Rey et al., 2017). Therefore, Rey et al. (2017) proposed that GRXS14 may regulate the redox status of chlorophyll biosynthetic enzymes and/or participate in the assembly and transfer of 2Fe-2S in the plastid, especially under stress conditions. A number of enzymes involved in chlorophyll metabolism contain Fe-S clusters as cofactors: chlorophyllide *a* oxygenase (CAO, EC 1.14.13.122), 7-hydroxymethyl chlorophyll *a* reductase (HCAR, EC 1.17.7.2), and pheophorbide *a* oxygenase (PAO, EC 1.14.15.17) (Oster et al., 2000; Pruzinská et al., 2003; Meguro et al., 2011). Plastidial GRXs may control the redox status of chlorophyll biosynthetic enzymes by regulating the assembly and transfer pathway of Fe-S clusters in the plastids. Consistent with this hypothesis, GRXs, including GRXS14 and GRXS16, could interact with the BolA domain of SufE1 (Table 2) and thereby deglutathionate SufE1, which facilities the activation of plastidial Cys desulfurase (Couturier et al., 2014).

GRXS16 has an N-terminal GlyIleTyr-TyrIleGly (GIY-YIG) endonuclease domain (Figure 3), which confers GRXS16 unique functions, such as coordinating redox regulation and DNA cleavage (Liu et al., 2013). The GIY-YIG domain of GRXS16 alone has endonuclease activity and the activity is decreased substantially in GRXS16 (Liu et al., 2013). On the other hand, the presence of the GIY-YIG domain reduces the ability of GRXS16 to suppress the susceptibility of yeast *grx5* mutant to oxidative stress. These data suggest that the two functional domains in GRXS16 negatively regulate each other. Furthermore, Liu et al. (2013) found that Cys^123^ in the GIY-YIG domain may form an intramolecular disulfide bond with Cys^219^ in the CGFS motif of the GRX domain, which explains the negative regulation between the two functional domains.

#### At-NEET

As mentioned previously, At-NEET has a CDGSH motif (Figure 3; Table 1), which contains the three Cys and one His residues necessary for NEET-type 2Fe-2S coordination (Nechushtai et al., 2012; Su et al., 2013). The first two Cys residues (C^74^ and C^76^) have been shown to be essential for cluster coordination (Su et al., 2013), while the His residue (H^89^) has been shown to be essential for the transferability of the 2Fe-2S cluster (Nechushtai et al., 2012). At-NEET forms homodimers (Table 2) and each homodimer coordinates two labile 2Fe-2S clusters, which are readily transferred to apo Fd in *in vivo* assays (Nechushtai et al., 2012). Knockdown and RNAi lines of At-NEET showed delayed growth and development, accelerated senescence, and elevated ROS accumulation under standard conditions, increased sensitivity to low Fe conditions, and reduced sensitivity to high Fe conditions (Nechushtai et al., 2012). These data suggest that At-NEET is important in Fe metabolism, ROS homeostasis, as well as plant growth, development, and senescence. Therefore, At-NEET might be a carrier protein for the assembly and transfer of NEET-type 2Fe-2S clusters (Nechushtai et al., 2012).

## Regulation of the plastidial Fe-S assembly and transfer pathway

Due to the toxicity of free Fe, the biosynthesis of Fe-S clusters has to be regulated together with Fe uptake (Balk and Schaedler, 2014). Hierarchical clustering analysis of Arabidopsis genes involved in Fe-S assembly and transfer pathways with publicly available microarray data showed that genes for the plastidial SUF pathway form one cluster and genes for the mitochondrial ISC pathway and the cytosolic CIA pathway form another (Balk and Schaedler, 2014). For example, the *SufA1, SufB, SufC, SufD*, and *SufE1* genes cluster together, possibly due to their high expression in photosynthetic tissues (Balk and Schaedler, 2014). These observations suggest that the expression of *SUF* genes is coordinated and that the plastidial SUF pathway and the ISC/CIA pathways might be differentially regulated. The latter is consistent with the relative independency of the SUF pathway from the ISC/CIA pathways (Van Hoewyk et al., 2007; Bernard et al., 2013).

Balk and Schaedler (2014) also found that under pathogen infection (*Pseudomonas syringae*) or oxygen deficiency, the expression of *ISC* and *SUF* genes is down-regulated (Balk and Schaedler, 2014). Following recognition of the pathogen, the host plant immediately up-regulates ROS production, as part of the defense responses (Torres et al., 2006; Torres, 2010). The rapid rise in ROS tends to destroy Fe-S clusters (Balk and Schaedler, 2014). Therefore, down-regulation of Fe-S cluster assembly at the transcriptional level could be viewed as a preventive method to minimize the content of toxic free Fe released from Fe-S clusters (Balk and Schaedler, 2014).

Several studies showed that *SufB* gene expression in Arabidopsis is down-regulated under Fe-deficient conditions (Xu et al., 2005; Balk and Schaedler, 2014). This trend was also observed in rice (Liang et al., 2014). Because SufB is an essential Fe-S scaffold protein (Nagane et al., 2010), a decrease in *SufB* gene expression may quickly decrease the overall rate of Fe-S synthesis in the plastid (Balk and Schaedler, 2014). Further studies are needed to understand the molecular mechanism for Fe deficiency-induced down-regulation of *SufB* gene expression.

Among Fe-S assembly-related genes, *SufE2* was very responsive to a wide range of environmental stimuli (Balk and Schaedler, 2014). As mentioned above, SufE2 only has a single SufE domain, unlike SufE1 and SufE3, which have an additional BolA and NadA domain, respectively (Murthy et al., 2007). Therefore, Balk and Schaedler (2014) proposed that SufE2 may regulate the flux of persulfide (R-S-S^0^H) groups between BolA, the C-terminal domain of SufE1, and NadA, the C-terminal domain of SufE3.

As mentioned above, the two plastid-targeted GRX proteins, GRXS14 and GRXS16, have two Fe-S assembly-related roles. On one hand, GRXS14 and GRXS16 serve as Fe-S carriers in the plastidial Fe-S assembly and transfer pathway (Bandyopadhyay et al., 2008). On the other hand, GRXS14 and GRXS16 are important regulators of redox homeostasis inside the plastid (Rey et al., 2017). GRXS14 and GRXS16 were found to interact with the BolA domain of SufE1 (Couturier et al., 2014). These observations indicate that SufE1 activity and overall Fe-S cluster assembly in the plastid may be subject to redox regulation by GRXS14 and GRXS16 (Couturier et al., 2014). In addition, the interactions between the SufE1 BolA domain and the two plastidial GRX proteins suggest potential link or regulation between the upstream persulfide (R-S-S^0^H) flux at the SufSE complex and the downstream Fe-S transfer steps via Fe-S carrier proteins.

Another protein with a potential regulatory role is At-NEET, a NEET-type 2Fe-2S-containing protein dually targeted to plastids and mitochondria (Nechushtai et al., 2012; Su et al., 2013). As mentioned in a previous paragraph, knockdown and RNAi of At-NEET expression resulted in delayed growth and developmental retardation, enhanced senescence, and increased ROS under standard conditions (Nechushtai et al., 2012). The same mutants also displayed elevated sensitivity to low Fe and decreased sensitivity to high Fe (Nechushtai et al., 2012). Therefore, Nechushtai et al. (2012) concluded that At-NEET is important in Fe metabolism and ROS homeostasis. Additional studies are needed to investigate whether and how At-NEET plays a role in regulating Fe-S assembly.

## Concluding remarks and outstanding questions

Fe-S clusters and proteins are essential to many biological processes. They are found in the plastids, mitochondria, cytosol, and nucleus of plant cells. Plastidial Fe-S clusters are assembled by the SUF pathway, which consists of Cys desulfurase NFS2, sulfur transferases SufE1, SufE2, and SufE3, SufBC_2_D scaffold complex, and carrier proteins. Cys is the S source for *in vivo* assembly of Fe-S clusters. However, it is not yet known what the *in vivo* Fe source is and how Fe is delivered to the scaffold complex. The NFS2-SufE1/2/3-SufBC_2_D system is responsible for *de novo* assembly of all plastidial Fe-S clusters, because no other Fe-S cluster assembly pathway has been found in the plastid. Single loss-of-function mutations of the NFS2-SufE1/2/3-SufBC_2_D system often result in an embryo- or seedling-lethal phenotype, suggesting that the NFS2-SufE1/2/3-SufBC_2_D system is essential. After assembly, Fe-S clusters need to be delivered to appropriate recipient proteins via carrier proteins. A total of nine potential plastidial Fe-S carriers have been identified thus far: SufA1, NFU1, NFU2, NFU3, HCF101, COG0354p, GRXS14, GRXS16, and At-NEET. SufA1, NFU2, GRXS14, and GRXS16 have been shown to act as carrier proteins transferring classic 2Fe-2S from the SufBC_2_D scaffold complex to recipient proteins. NFU3 has been shown to act as the carrier protein for 3Fe-4S. NFU2, NFU3, and HCF101 have been shown to act as 4Fe-4S carriers. At-NEET is a possible carrier protein for NEET-type 2Fe-2S.

Although NFU1 is a functional NFU protein, it is not clear which Fe-S clusters are transferred by this protein. It is possible that NFU1 plays a role in transferring 2Fe-2S, 3Fe-4S, and/or 4Fe-4S clusters because this protein contains a redox-active NFU domain with a conserved CXXC motif capable of coordinating 2Fe-2S, 3Fe-4S, and 4Fe-4S clusters. Functional analysis of recombinant NFU1 protein as well as immunodetection and activity assays of representative plastidial Fe-S proteins in loss-of-function *nfu1* mutants will help address these questions. The types of Fe-S clusters COG0354p transfers are also elusive. It is possible that COG0354p plays a role in transferring 4Fe-4S and classic 2Fe-2S clusters, because inactivation of COG0354 family protein IBA57 reduces the activities of 4Fe-4S and classic 2Fe-2S-containing enzymes.

AT-NEET is listed as a carrier protein for NEET-type 2Fe-2S. The recombinant AT-NEET homodimer contains two labile 2Fe-2S clusters, which can be transferred to apo Fd *in vitro* (Nechushtai et al., 2012). However, whether plant-type Fds act as an *in vivo* recipient protein for NEET-type 2Fe-2S from AT-NEET is still questionable, because plant-type Fds are known as classic 2Fe-2S proteins. Additional experiments are necessary to identify the *in vivo* recipient proteins for NEET-type 2F-2S and to test whether AT-NEET is capable of transferring NEET-type 2Fe-2S to recipient proteins under *in vivo* conditions.

It is not yet known which plastidial carriers are responsible for transferring Rieske-type 2Fe-2S to recipient proteins. NFU1 does not seem to be a good candidate because it does not contain the appropriate binding domain (CXHXGCX_12−44_CXCH) for Rieske-type 2Fe-2S. Although it is well-established that GRXS14 and GRXS16 transfer classic 2Fe-2S, it is possible that these two GRXs may transfer other Fe-S clusters as well. The expression of plastidial GRXS14 and GRXS16 in yeast *grx5* mutant restores the activity of mitochondrial ACO, whose function requires 4Fe-4S. These results suggest that GRXS14 and GRXS16 may act as carrier proteins for 4Fe-4S as well. Functional analysis of recombinant GRXS14 and GRXS16 as well as immunodetection and activity assays of representative plastidial 4Fe-4S proteins in loss-of-function *grxs14* and *grxs16* mutants will help test this hypothesis.

3Fe-4S and 4Fe-4S have similar absorption spectra, with a broad peak around 410 nm (Kennedy et al., 1984; Nakamaru-Ogiso et al., 2002). Therefore, it is challenging to distinguish the specific types of Fe-S clusters transferred by a Fe-S carrier protein simply via absorption spectral characterization of as-purified and reconstituted Fe-S carrier proteins. Immunodetection and activity assays of representative plastidial proteins for each type of Fe-S clusters in loss-of-function mutants will provide complementary information.

Furthermore, the unidirectional and intact transfer of Fe-S clusters from one carrier to another raises the possibly that some Fe-S carriers may work together in a sequence when delivering Fe-S clusters to the appropriate recipient proteins.

Lastly, although several possible regulation routes have been proposed for the SUF pathway, it is a relatively understudied area. Further investigations are needed to gain a more holistic understanding of the regulatory network of Fe-S assembly and transfer pathways in plants.

## Author contributions

The author confirms being the sole contributor of this work and approved it for publication.

### Conflict of interest statement

The author declares that the research was conducted in the absence of any commercial or financial relationships that could be construed as a potential conflict of interest.

## Abbreviations

ABA: abscisic acid deficient 3
ABC: ATP binding cassette
ACO: aconitase
APR1: adenosine 5′-phosphosulfate reductase 1
At: *Arabidopsis thaliana*
ATPase: adenosine triphosphatase
BIO: biotin synthase
BolA: DNA-binding transcriptional regulator BolA
CAO: chlorophyllide a oxygenase
CIA: cytosolic iron–sulfur pathway
COG0354m and COG0354p: mitochondrial and plastidial Clusters of Orthologous Groups 0354 proteins
CpNifS: chloroplastic nitrogen fixation S-like
CpIscA1: chloroplast iron–sulfur cluster protein A1
CpSufS: chloroplastic sulfur mobilization protein S
DUF59 and DUF971: domain of unknown function 59 and 971
Fd: ferredoxin
Fd-GOGAT1 and Fd-GOGAT2: ferredoxin-dependent Gln oxoglutarate aminotransferase 1 and 2
FER1, FER2, and FER3: ferritin 1, 2, and 3
FNR: fumarate and nitrate reduction regulatory protein
FTR: ferredoxin-thioredoxin reductase
GFP: green fluorescent protein
GIY-YIG: GlyIleTyr-TyrIleGly
GRX: glutaredoxin
GRXS14 and GRXS16: glutaredoxin S14 and S16
HCAR: 7-hydroxymethyl chlorophyll *a* reductase
HCF101: high chlorophyll fluorescence 101
IRP1: iron regulatory protein 1
ISC: iron–sulfur cluster pathway
IscS: iron–sulfur cluster protein S
ISU1: iron sulfur cluster assembly protein 1
LEU1: 3-isopropylmalate isomerase 1
LIP: lipoic acid synthase
MiaB: isopentenyl-adenosine A37 tRNA methylthiolase
NadA: quinolinate synthase
NAP1, NAP6, and NAP7: non-intrinsic ABC protein 1, 6, and 7
NFU1, NFU2, and NFU3: nitrogen fixation subunit U 1, 2, and 3
NFS1/NifS1 and NFS2: nitrogen fixation S-like 1 and 2
NiR: nitrite reductase
NTPase: nucleotide phosphatase
PetC: photosynthetic electron transfer C
PAO: pheophorbide *a* oxygenase
PSI: Photosystem I
PsaA, PsaB, and PsaC: Photosystem I proteins A, B, and C
RNAi: RNA interference
SiR: sulfite reductase
SUF: sulfur mobilization pathway
SufA1, SufB, SufC, SufD, SufE1, SufE2, and SufE3: sulfur mobilization protein A1, B, C, D, E1, E2, and E3
TP: transit peptide
YFP: yellow fluorescent protein.
